# Supplementary material for: Competing risks models with two time scales
Source: Stat Methods Med Res. 2025 Sep 1;34(11):2145–62. doi: 10.1177/09622802251367443 (PMC12669410; doi:10.1177/09622802251367443)
Supplement: sj-pdf-1-smm-10.1177_09622802251367443 - Supplemental material for Competing risks models with two time scales [file sj-pdf-1-smm-10.1177_09622802251367443.pdf]

Competing risks models with two time scales
Supplementary material

1 SEER data analyzed in Section 3, descriptive statistics

	Age at diagnosis<br>Median	Deaths<br>Breast cancer	Deaths<br>Other causes	No. of deaths<br>Total
White, luminal A, no chemotherapy	67	6627 (35.3)	12173 (64.7)	18800 (19.7)
White, luminal A, chemotherapy	60	4396 (74.8)	1483 (25.2)	8792 (29.1)
White, other subtypes, no chemotherapy	70	2291 (53.3)	2009 (46.7)	4300 (36.7)
White, other subtypes, chemotherapy	61	4333 (75.6)	1401 (24.4)	5734 (22.2)
Black, luminal A, no chemotherapy	66	961 (43.5)	1248 (66.5)	2209 (25.4)
Black, luminal A, chemotherapy	59	943 (74.9)	316 (25.1)	1259 (14.5)
Black, other subtypes, no chemotherapy	67	517 (59.3)	355 (40.7)	872 (43.3)
Black, other subtypes, chemotherapy	59	1280 (78.8)	344 (21.2)	1624 (30.2)
Other, luminal A, no chemotherapy	65	542 (39.7)	824 (60.3)	1366 (13.6)
Other, luminal A, chemotherapy	59	466 (79.5)	120 (20.5)	586 (16.4)
Other, other subtypes, no chemotherapy	65	223 (58.1)	168 (41.9)	391 (26.1)
Other, other subtypes, chemotherapy	59	487 (81.4)	111 (18.6)	598 (17.1)

Supplementary Table 1: SEER data, descriptive statistics by subgroup. From left to right: Median age at diagnosis, number of breast cancer deaths (% of all deaths), number of deaths for other causes (% of all deaths), total number of deaths (% of all individuals in subgroup).

2 Correction for year 2020 follow-up

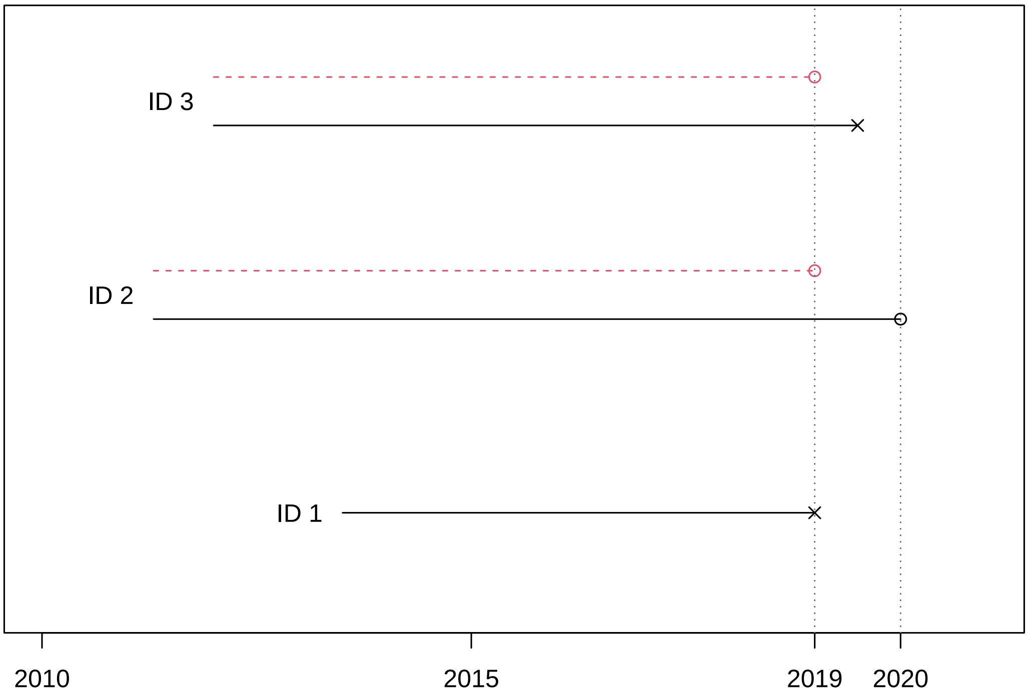

Supplementary Figure 1: Illustration of the adjustment of the follow-up time for individuals with last follow-up in 2020. Three hypothetical patients are pictured: the black lines represent the complete follow-up time as reported in the registers. The red broken lines represent the adjusted follow-up time at the end of 2019. Crosses indicate a death and empty circles indicate a right-censored observation. For individual 1 no correction is needed, while individuals 2 and 3 follow-up time is adjusted of 12 and 6 months respectively.

3 Cumulative Incidence Functions: Sampling distribution, bootstrap

Confidence intervals for the cumulative incidence functions are determined by bootstrapping, see Section 2.3 and 3.3 of the main paper. To support the approach, we investigated the estimation results by a simulation study. The cause-specific hazards mimic the shapes that resulted in the analysis of the SEER data. The cause-specific hazards and the corresponding cumulative incidence functions are shown in Figure 2.

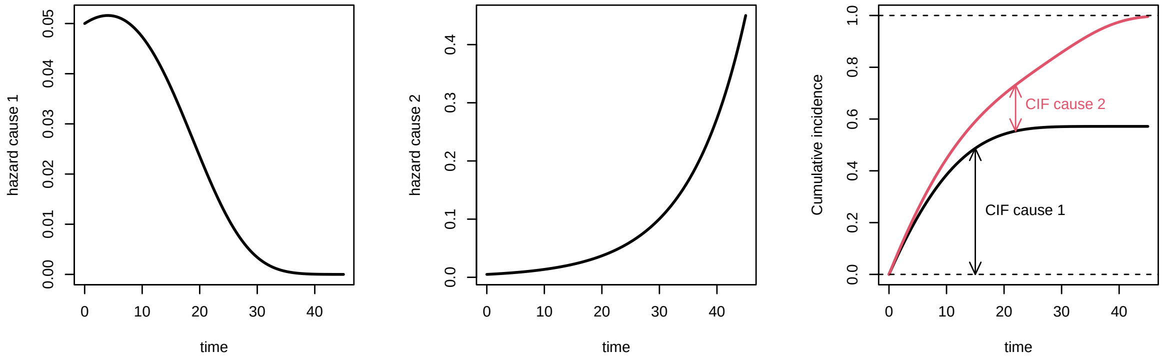

Supplementary Figure 2: Cause-specific hazards (left and center) and resulting cumulative incidence functions (right) of the simulation study.

We simulated data from this model (sample size $n = 500$ and did 500 replications. We also studied $n = 1000$ and $n = 3000$, but all sample sizes lead to the same assessment, so we present only a summary for $n = 500$). Observations were right-censored at time $s = 40$.

Figure 3 shows the average estimated values of the two cumulative incidence functions, verifying unbiased estimation.

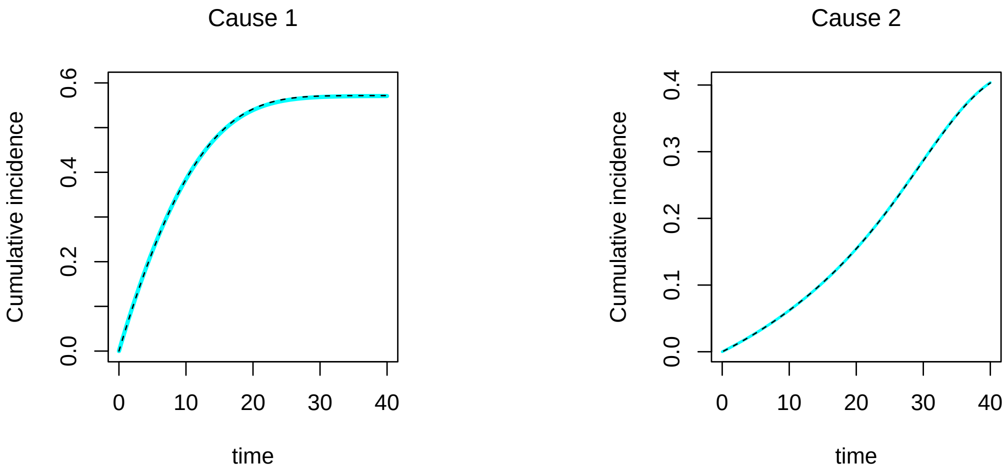

Supplementary Figure 3: Average of estimates (solid line) of cumulative incidence functions, 500 replications. Dashed line: True functions, see Fig.2.

To study the sampling distribution we present the Normal QQ-plots for both cumulative incidence functions at several time points in Figure 4. Consequently, we use the standard deviation from the bootstrap samples to estimate the standard errors of the cumulative incidence functions and calculate Normal-based confidence intervals, as described in Section 2.3 of the main paper.

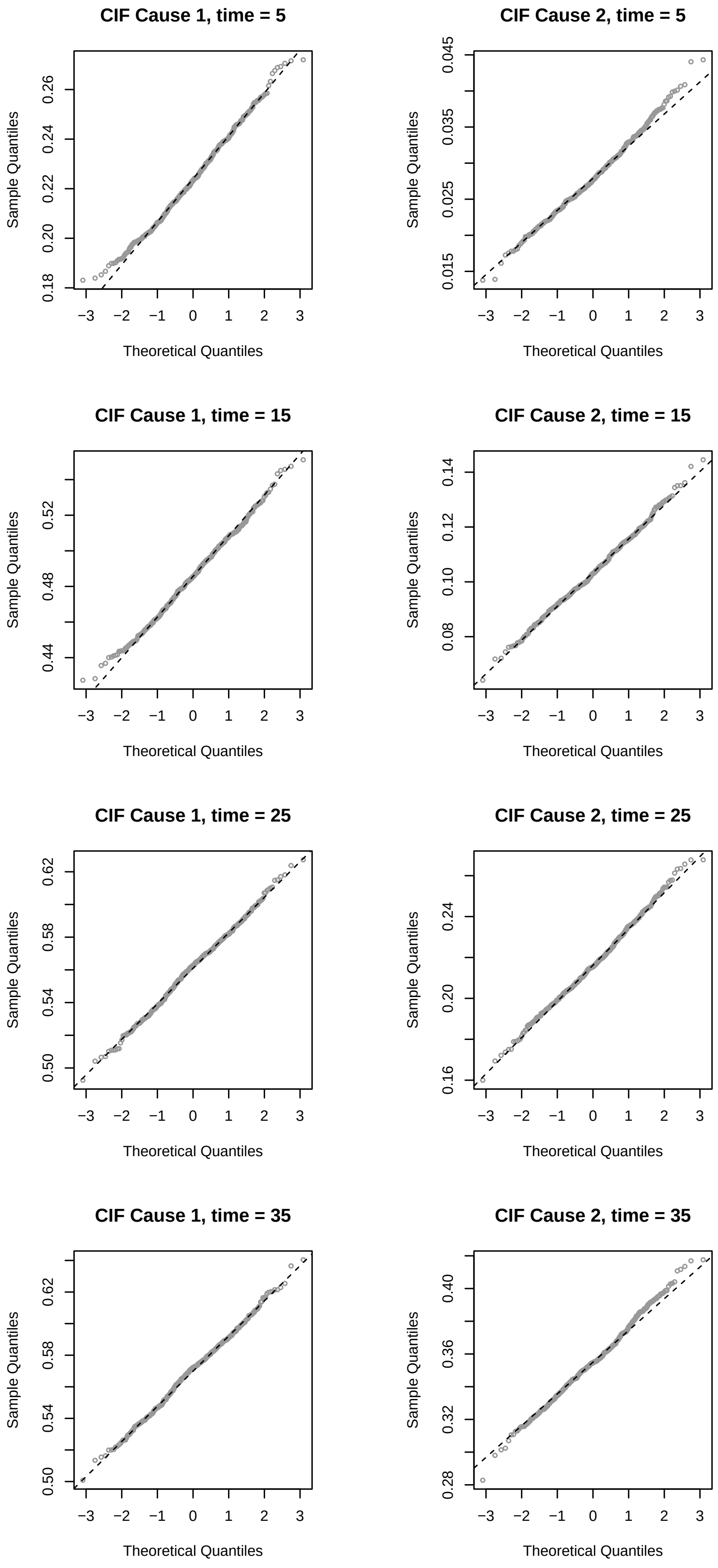

Supplementary Figure 4: Normal quantile-quantile plot of 500 estimates of the two cumulative incidence functions (left: cause 1, right: cause 2) at selected time points.

4 Estimation results, SEER data, all subgroups

	Death due to breast cancer			Other causes of death		
	$\log_{10} \varrho_u$	$\log_{10} \varrho_s$	ED	$\log_{10} \varrho_u$	$\log_{10} \varrho_s$	ED
White, other subtypes, no chemotherapy	2.5	1.7	7.2	6.6	5.8	4.0
White, other subtypes, chemotherapy	2.5	0.2	12.3	3.3	5.8	4.2
White, luminal A, no chemotherapy	2.0	5.6	7.9	2.6	2.3	9.4
White, luminal A, chemotherapy	2.3	1.3	9.6	2.3	1.8	6.4
Black, other subtypes, no chemotherapy	2.3	6.8	4.9	6.0	1.0	4.7
Black, other subtypes, chemotherapy	5.9	0.2	6.8	6.6	6.2	4.0
Black, luminal A, no chemotherapy	2.0	5.4	5.7	2.8	6.7	4.6
Black, luminal A, chemotherapy	2.1	1.3	6.6	5.2	4.6	4.0
Other, other subtypes, no chemotherapy	5.5	4.6	4.0	5.7	4.1	4.0
Other, other subtypes, chemotherapy	2.1	1.1	6.1	5.0	4.0	4.0
Other, luminal A, no chemotherapy	1.8	5.5	5.6	2.0	6.7	5.5
Other, luminal A, chemotherapy	6.1	0.7	5.3	1.6	6.6	4.6

Supplementary Table 2: Optimal smoothing parameters (minimal BIC) and resulting effective dimensions (ED) for cause-specific hazards in different subgroups, age at diagnosis $[50, 100)$.

5 Cause-specific hazards with/without final age group

To investigate the potential impact of ungrouping of the final age interval $[90, 100)$ by the PCLM (see Section 3) we estimate the cause-specific hazards both for ages at diagnosis [50, 100) and [50, 90), where in the latter case no ungrouping is required.

	Death due to breast cancer			Other causes of death		
	$\log_{10} \varrho_u$	$\log_{10} \varrho_s$	ED	$\log_{10} \varrho_u$	$\log_{10} \varrho_s$	ED
Age at diagnosis [50, 100)						
White, other subtypes, no chemotherapy	2.5	1.7	7.2	7.5	6.9	4.0
White, other subtypes, chemotherapy	2.5	0.2	12.3	3.3	6.5	4.2
Age at diagnosis [50, 90)						
White, other subtypes, no chemotherapy	2.2	1.8	7.0	5.6	5.7	4.0
White, other subtypes, chemotherapy	2.3	0.2	12.6	5.3	5.0	4.0

Supplementary Table 3: Optimal smoothing parameters (minimal BIC) and resulting effective dimensions (ED) for cause-specific hazards, age at diagnosis $[50, 100)$ (age interval $[90, 100)$ ungroupd by PCLM) and age at diagnosis $[50, 90)$ (no ungrouping required).

The corresponding figures of the estimated cause-specific hazards are shown in Supplementary Figures 5 and 6.

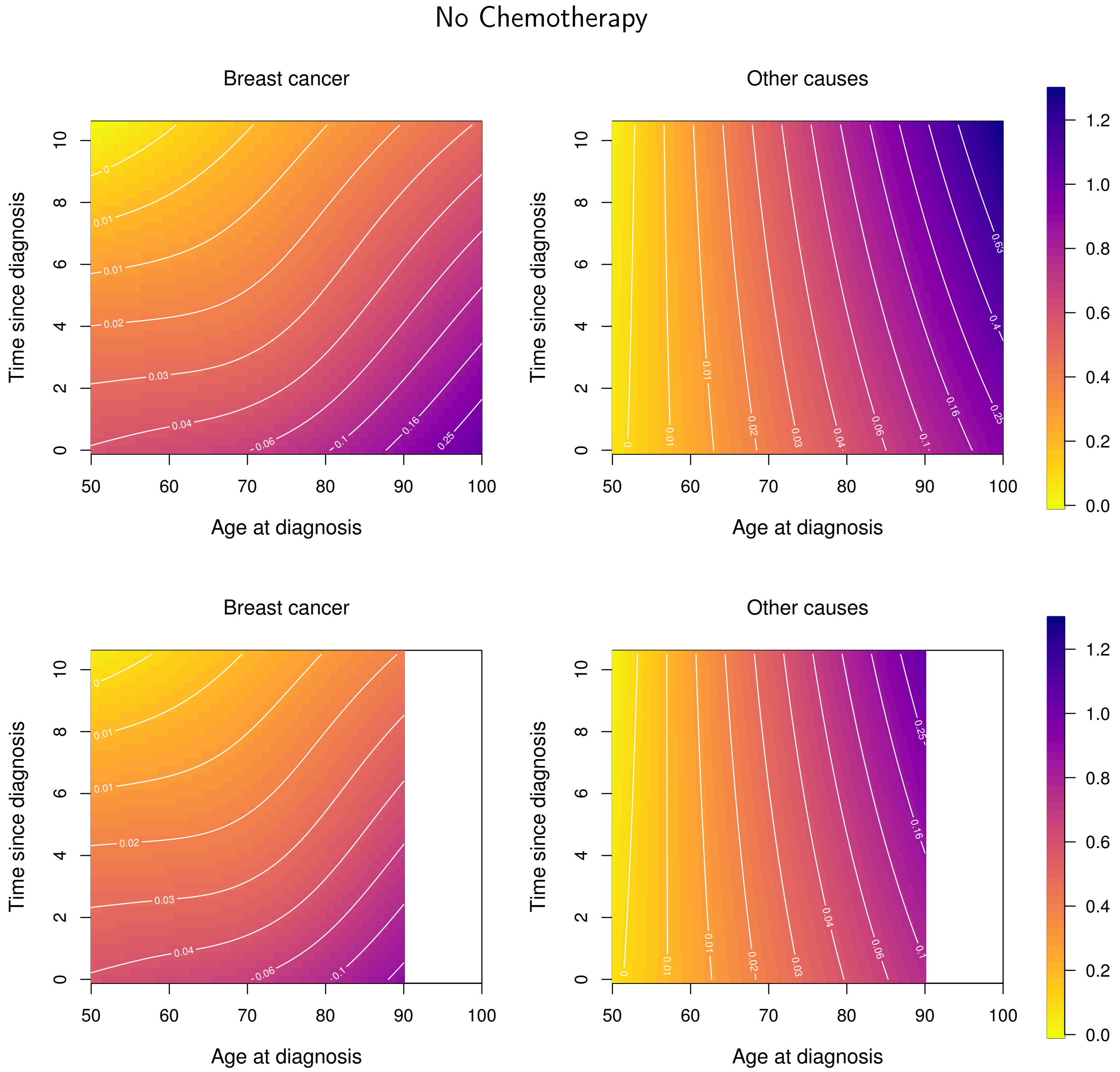

Supplementary Figure 5: Cause-specific hazards, white women, other subtypes, no chemotherapy. Top: Estimates based on ages at diagnosis [50, 100). Bottom: Estimates based on ages at diagnosis [50, 90). Smoothing parameters and effective dimensions are given in Supplementary Table 3.

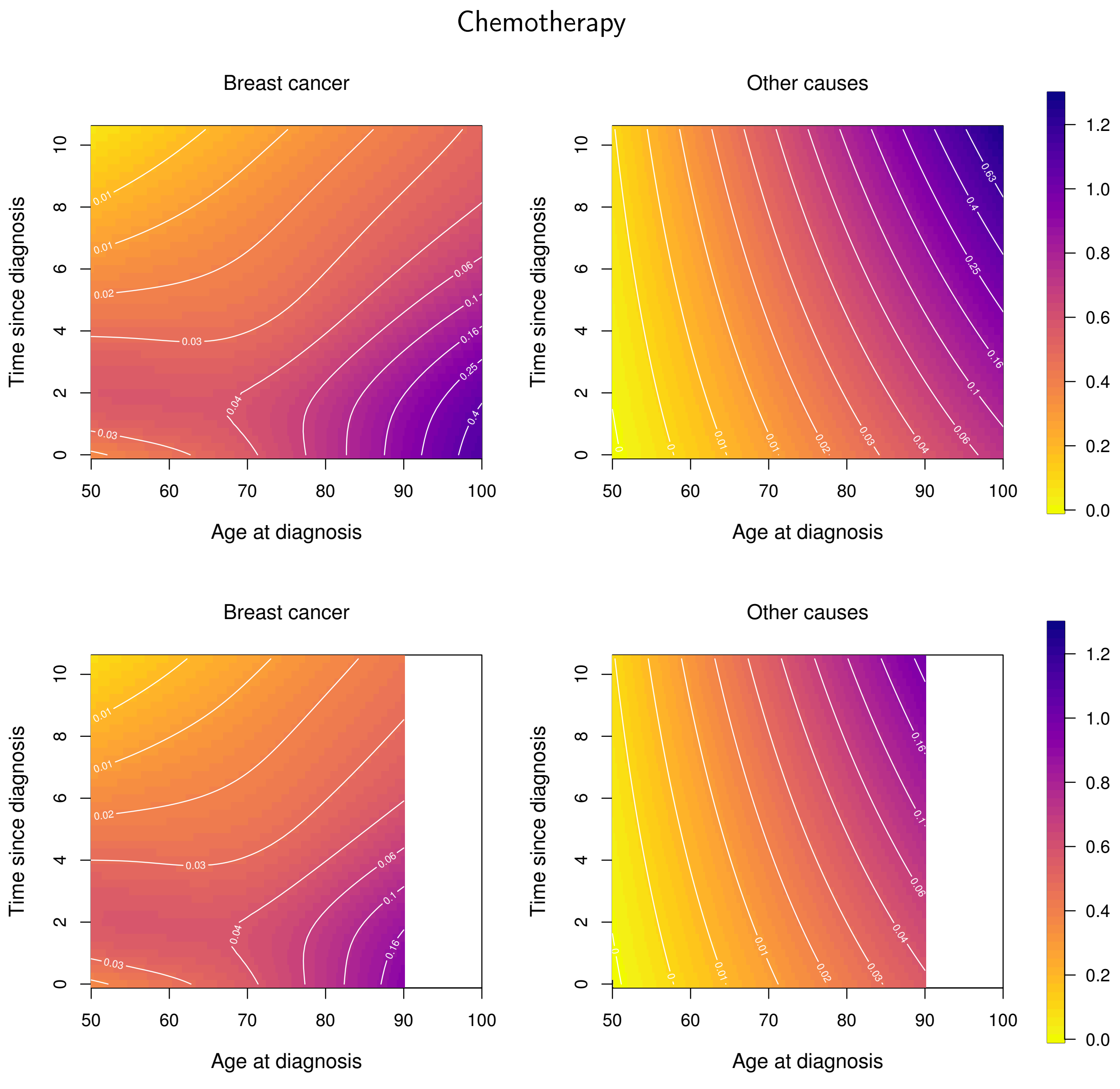

Supplementary Figure 6: Cause-specific hazards, white women, other subtypes, chemotherapy. Top: Estimates based on ages at diagnosis [50, 100). Bottom: Estimates based on ages at diagnosis [50, 90). Smoothing parameters and effective dimensions are given in Supplementary Table 3.

6 Additional results from the competing risks model for the SEER data

White women with luminal A subtype

Cause-specific hazards

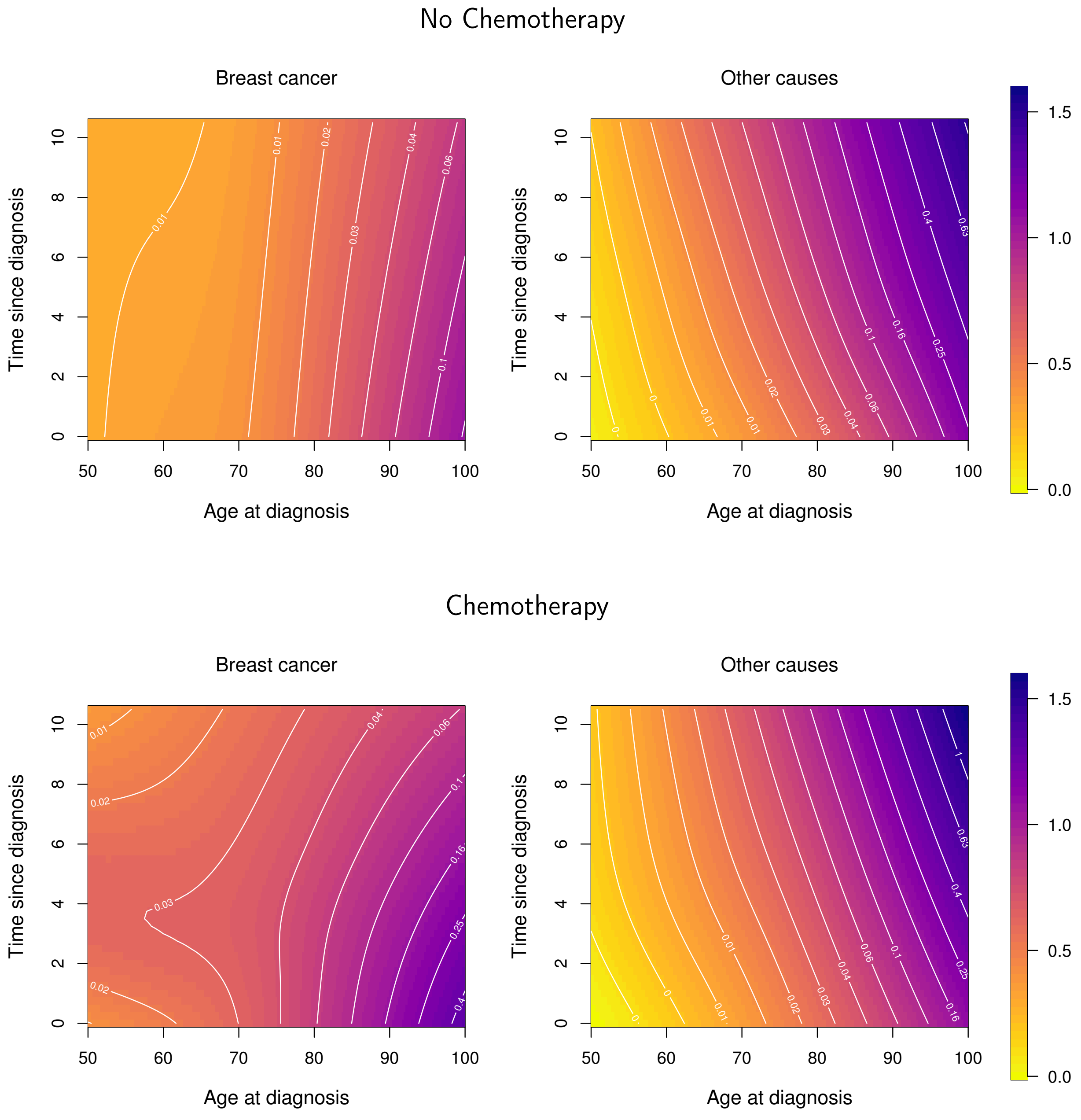

Supplementary Figure 7: Cause-specific hazards for white women, Luminal A cancer subtype who received chemotherapy (bottom row) and who did not (top row).

Cause-specific cumulative incidence

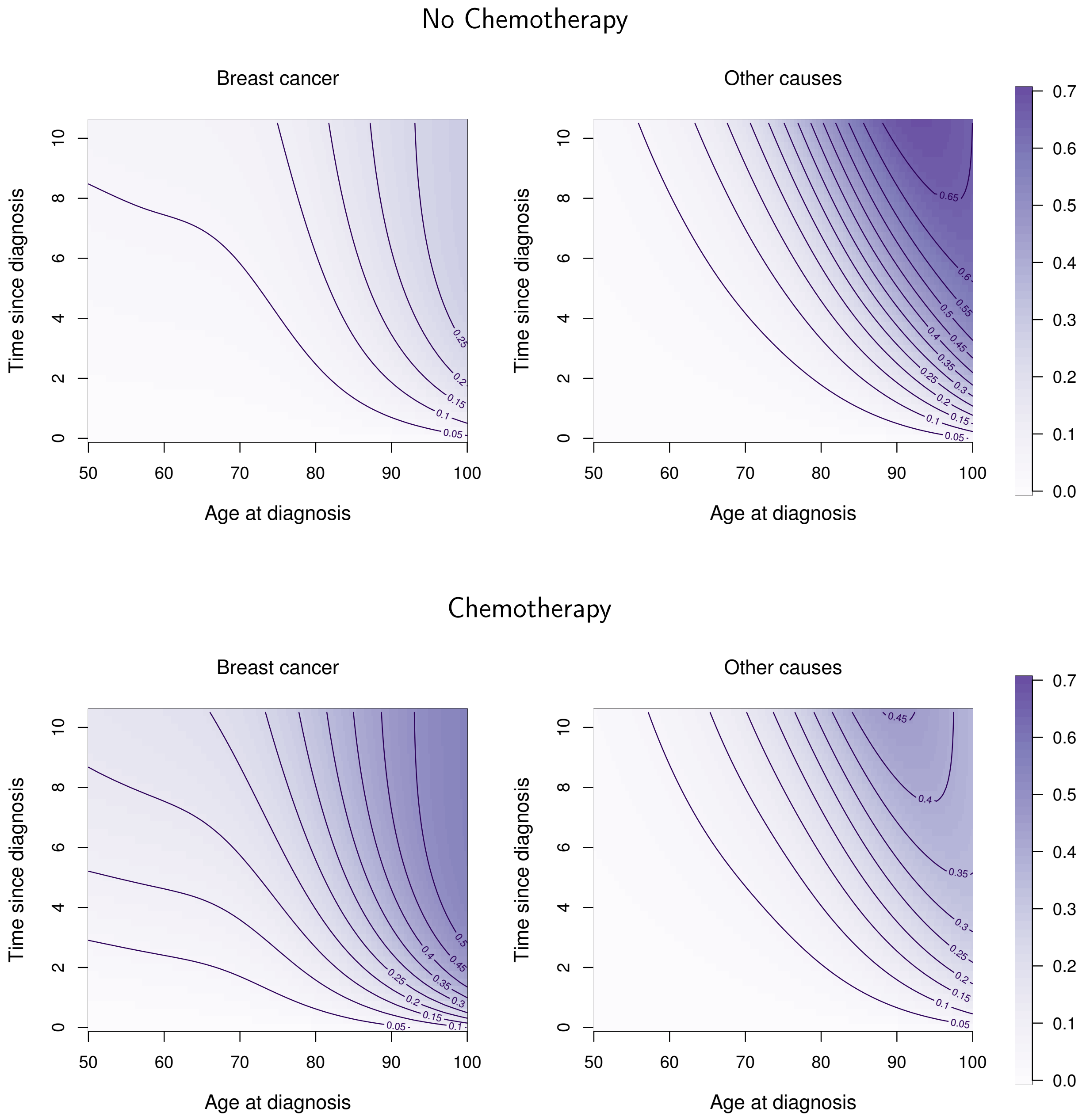

Supplementary Figure 8: Cause-specific cumulative incidence for white women, Luminal A cancer subtype who received chemotherapy (bottom row) and who did not (top row).

Black women with luminal A subtype

Cause-specific hazards

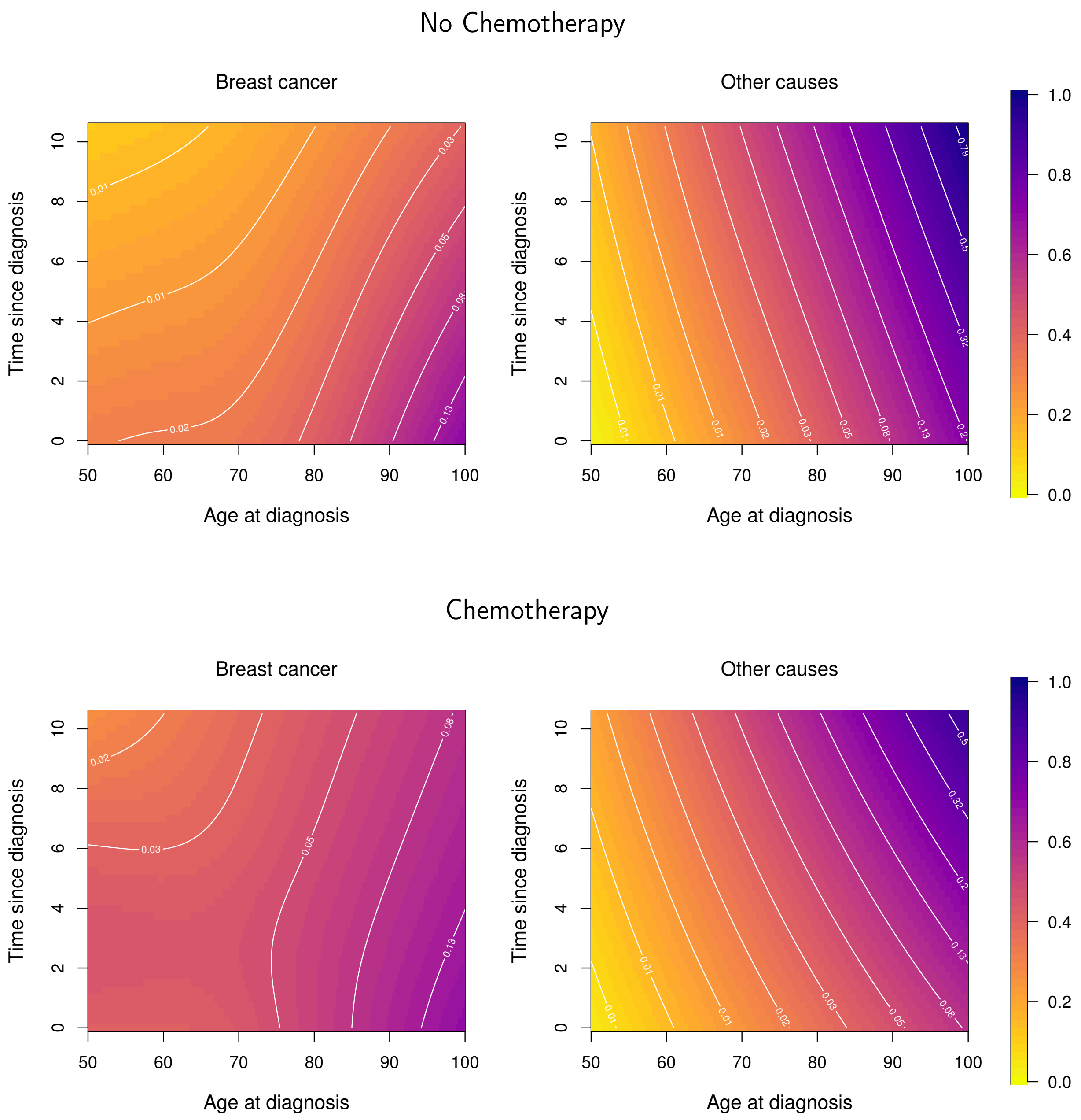

Supplementary Figure 9: Cause-specific hazards for black women, Luminal A cancer subtype who received chemotherapy (bottom row) and who did not (top row).

Cause-specific cumulative incidence

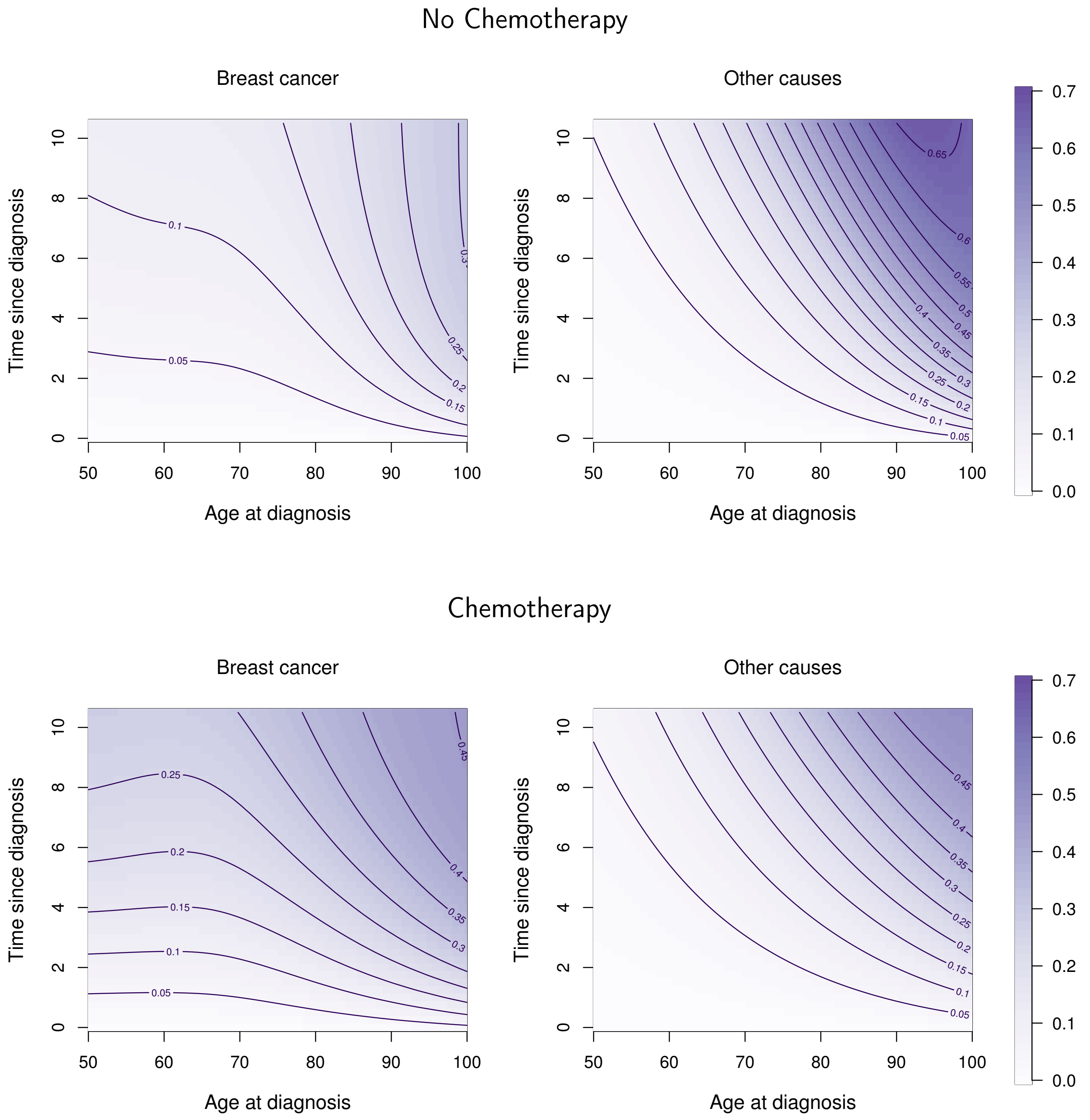

Supplementary Figure 10: Cause-specific cumulative incidence for black women, Luminal A cancer subtype who received chemotherapy (bottom row) and who did not (top row).

Black women with other subtypes

Cause-specific hazards

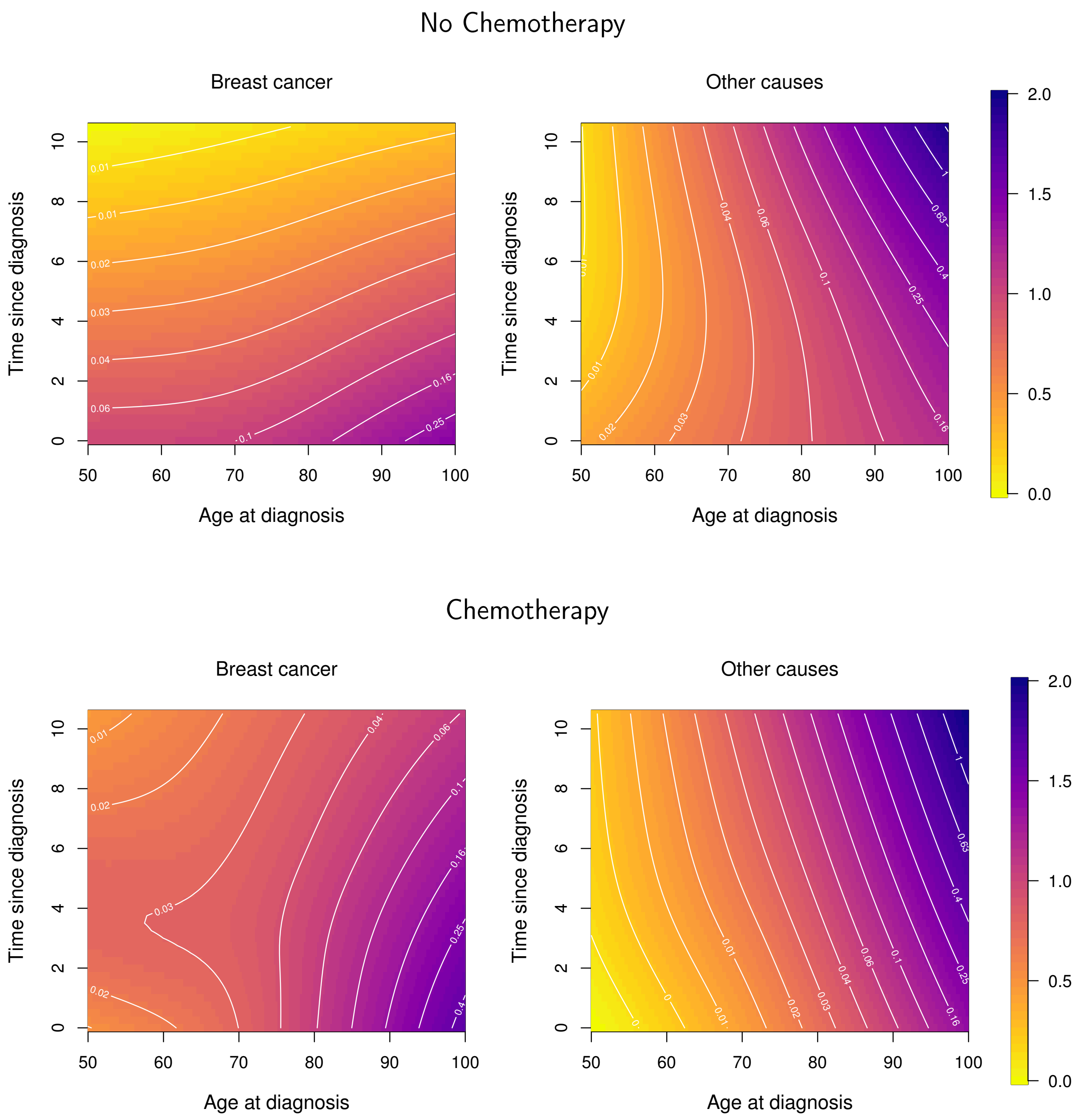

Supplementary Figure 11: Cause-specific hazards for black women, cancer subtypes other than Luminal A who received chemotherapy (bottom row) and who did not (top row).

Cause-specific cumulative incidence

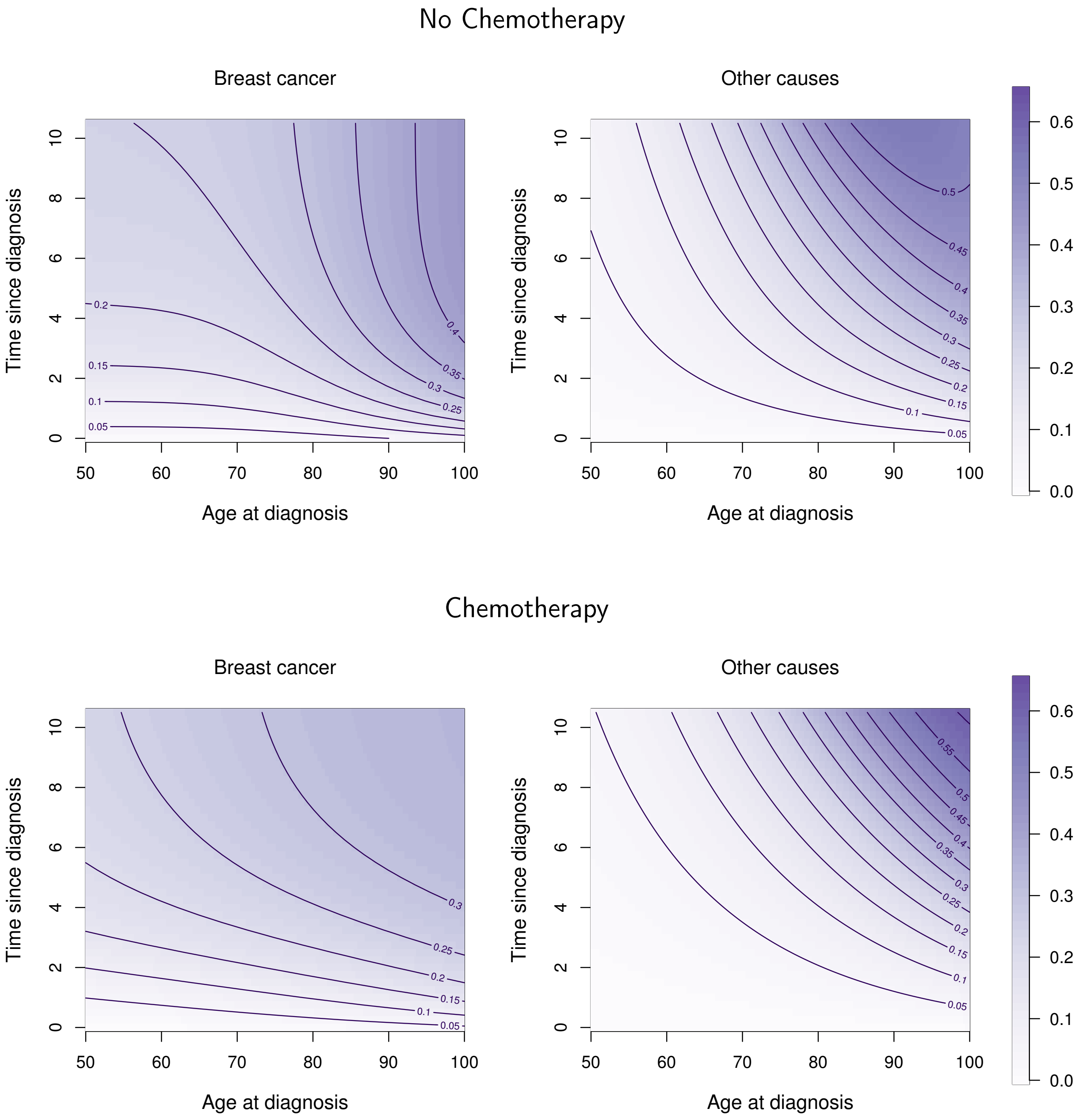

Supplementary Figure 12: Cause-specific cumulative incidence for black women, cancer subtypes other than Luminal A who received chemotherapy (bottom row) and who did not (top row).

Other race/ethnicity with luminal A subtype

Cause-specific hazards

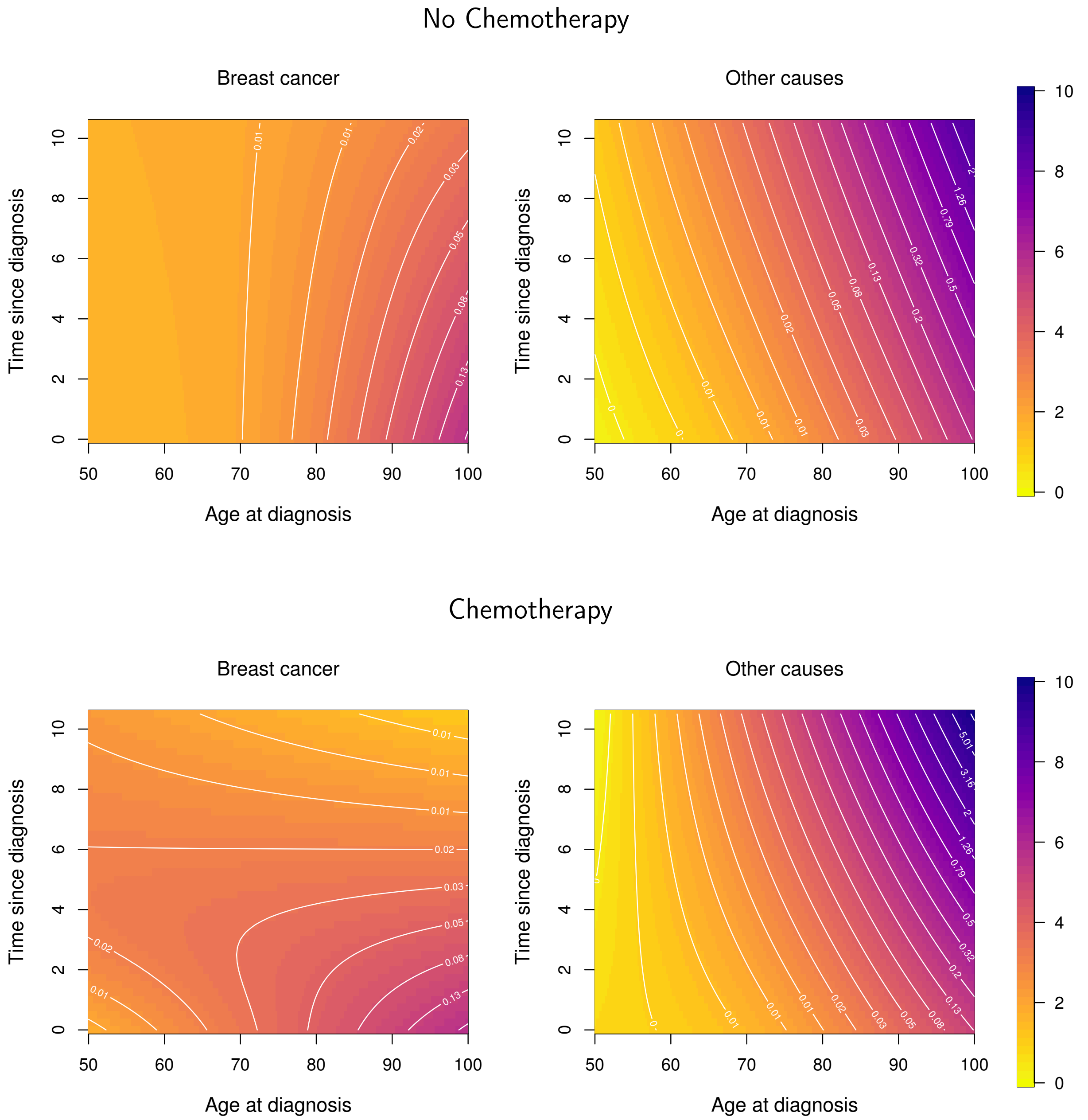

Supplementary Figure 13: Cause-specific hazards for women of race/ethnicity other than black or white, Luminal A cancer subtype who received chemotherapy (bottom row) and who did not (top row).

Cause-specific cumulative incidence

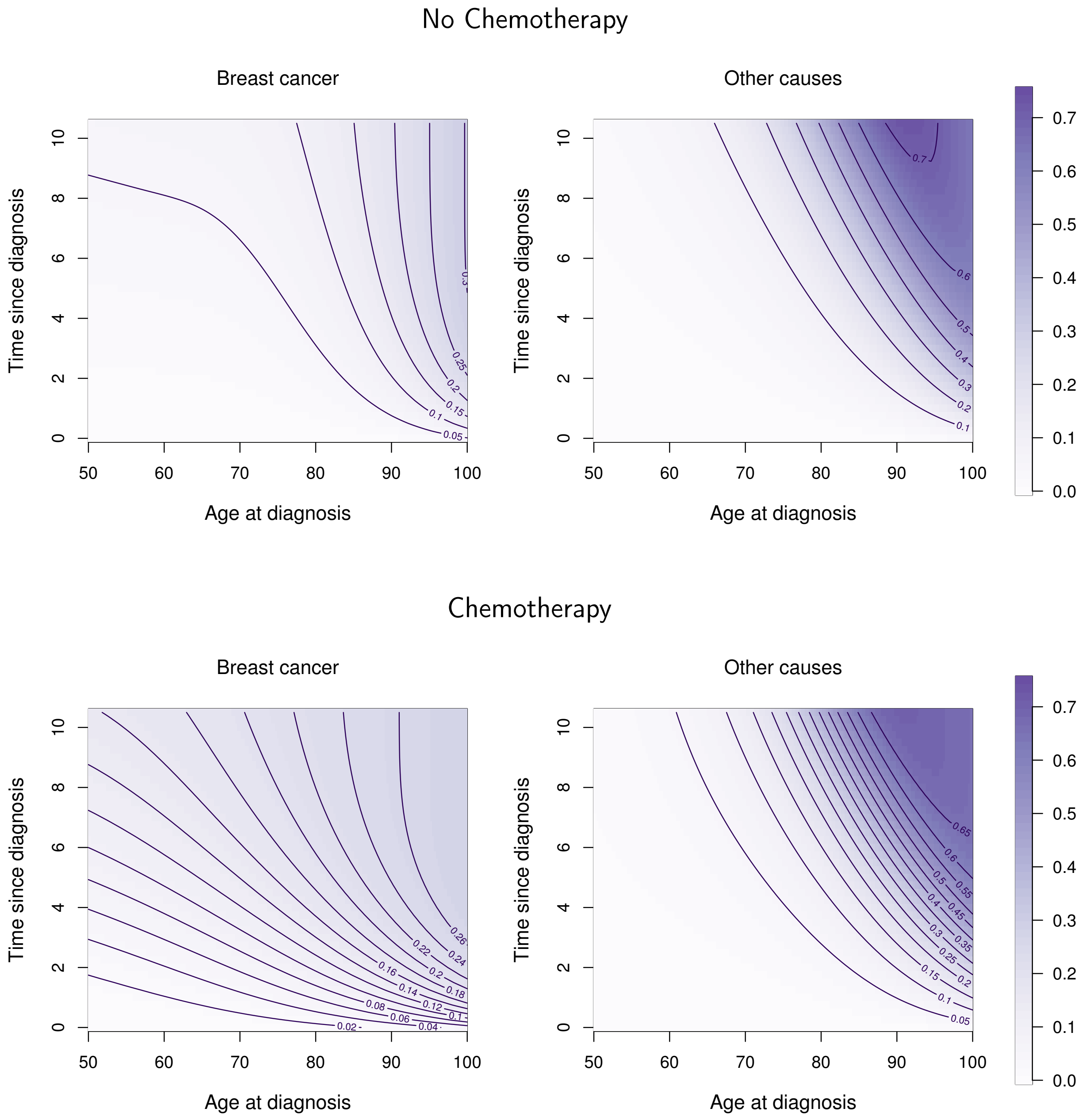

Supplementary Figure 14: Cause-specific cumulative incidence for women of race/ethnicity other than black or white, Luminal A cancer subtype who received chemotherapy (bottom row) and who did not (top row).

Other race/ethnicity with other subtype

Cause-specific hazards

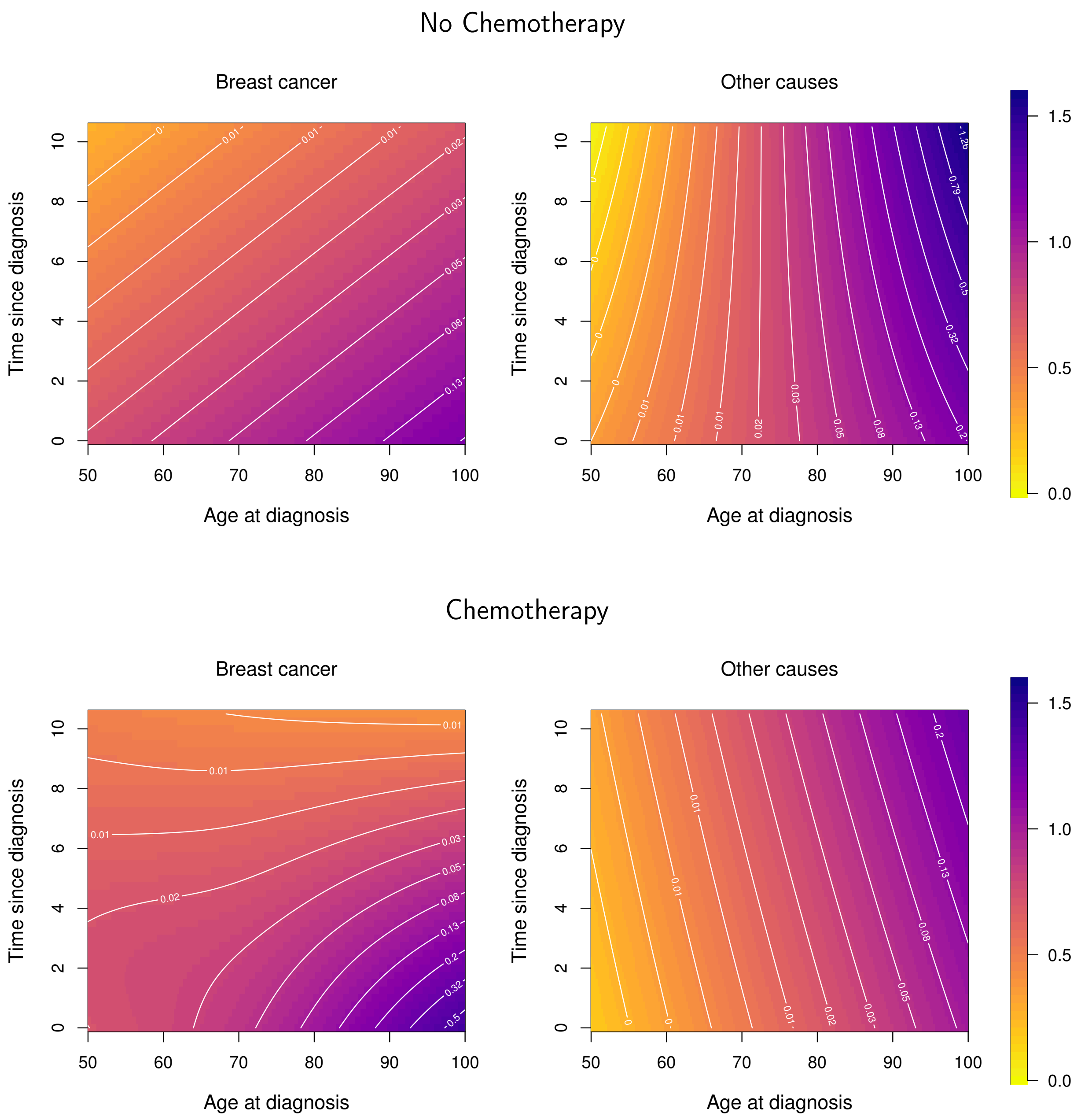

Supplementary Figure 15: Cause-specific hazards for women of race/ethnicity other than black or white, cancer subtypes other than Luminal A who received chemotherapy (bottom row) and who did not (top row).

Cause-specific cumulative incidence

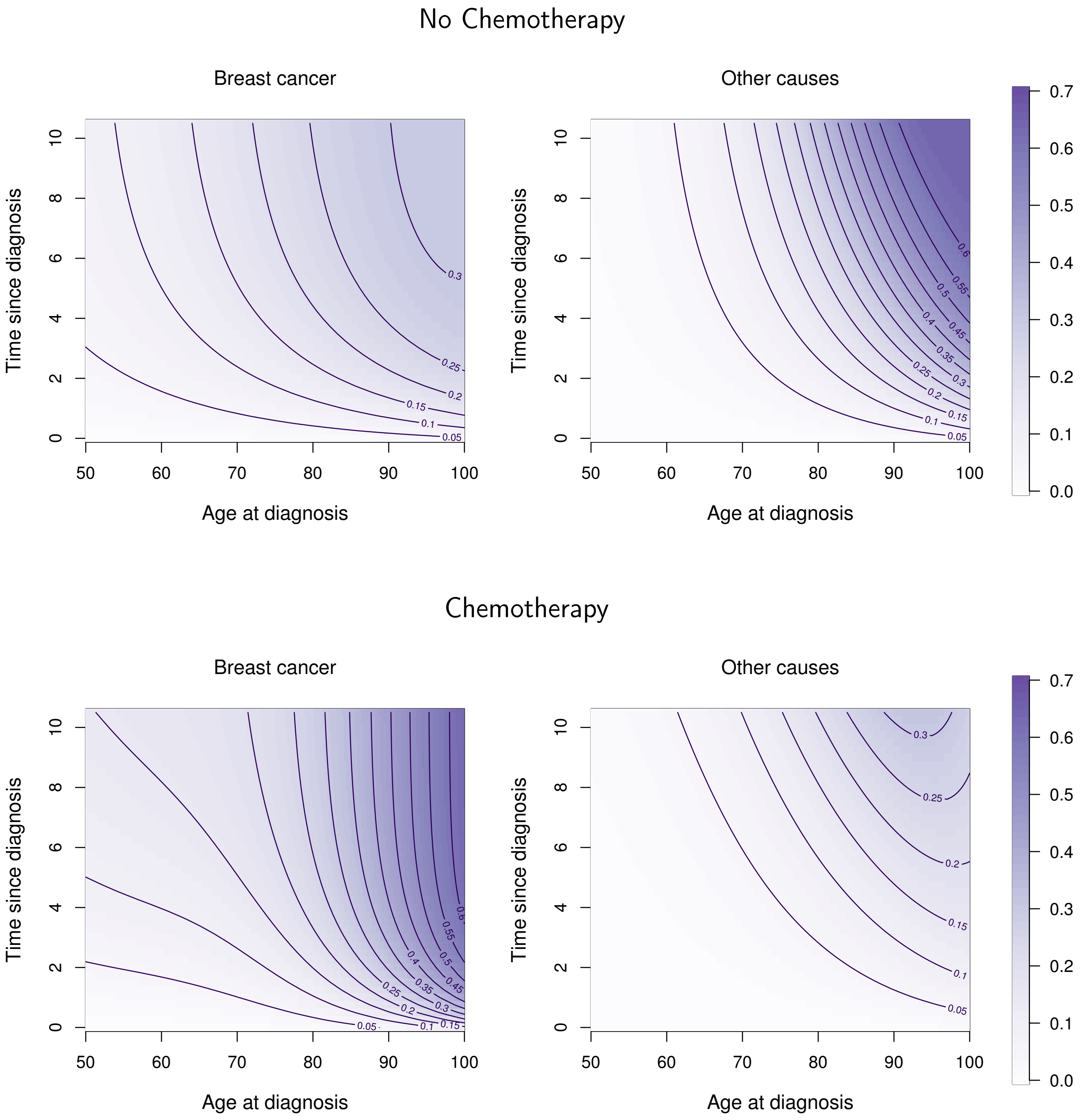

Supplementary Figure 16: Cause-specific cumulative incidence for women of race/ethnicity other than black or white, cancer subtypes other than Luminal A who received chemotherapy (bottom row) and who did not (top row).
